# Nanocarriers in Veterinary Medicine: A Challenge for Improving Osteosarcoma Conventional Treatments

**DOI:** 10.3390/nano12244501

**Published:** 2022-12-19

**Authors:** Simona Sapino, Giulia Chindamo, Daniela Chirio, Silvia Morel, Elena Peira, Cristina Vercelli, Marina Gallarate

**Affiliations:** 1 Dipartimento di Scienza e Tecnologia del Farmaco, Università degli Studi di Torino, 10125 Torino, Italy; 2 Dipartimento di Scienze del Farmaco, Università del Piemonte Orientale A. Avogadro, 28100 Novara, Italy; 3 Dipartimento di Scienze Veterinarie, Università degli Studi di Torino, 10095 Grugliasco, Italy

**Keywords:** veterinary medicine, canine osteosarcoma, nanocarriers, drug delivery, comparative oncology, animal model

## Abstract

In recent years, several nanocarrier-based drug delivery systems, such as polymeric nanoparticles, solid lipid nanoparticles, metallic nanoparticles, liposomes, and others, have been explored to target and treat a wide variety of diseases. Their employment has brought many benefits, not only to human medicine but also to veterinary medicine, albeit at a slower rate. Soon, the use of nanocarriers could revolutionize the animal health sector, and many veterinary therapies will be more effective as a result. The purpose of this review is to offer an overview of the main applications of nanocarriers in the veterinary field, from supplements for animal health and reproduction to nanovaccines and nanotherapies. Among the major pathologies that can affect animals, special attention is given to canine osteosarcoma (OSA): a comparison with human OSA is provided and the main treatment options are reviewed emphasizing the benefits that nanocarriers could bring in the treatment of this widespread disease.

## 1. Introduction

Nanotechnologies are widely known due to their broad range of applications in electronics, energy, environment, food, medicine, and consumer products. In recent decades, their application in human and veterinary medicine has primarily focused on the use of nanocarriers, such as nanoparticles (NPs), conjugates and other platforms, as drug delivery systems to increase the bioavailability of poorly soluble drugs and to protect unstable therapeutic agents from degradation. Due to their small size, nanocarriers can help overcome biological barriers enabling drugs to reach the site of action more easily. Furthermore, the modifiable surfaces of nanocarriers also expand their usability in targeted therapy [1,2].

**Figure 1:**
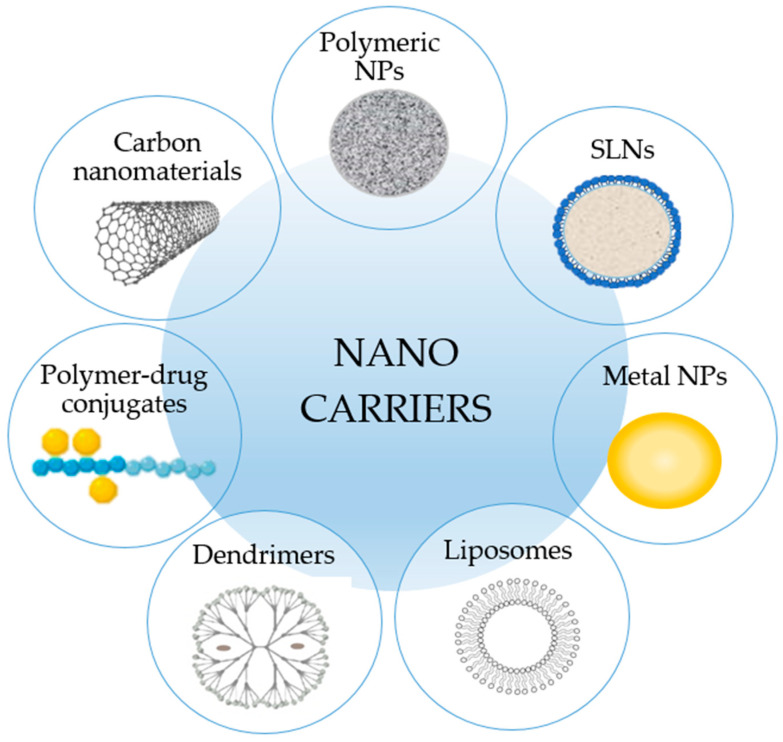
Schematic representation of the most important nanocarriers reported in veterinary literature.

Many classifications of nanocarriers exist based on their origin, shape, and applications. Among the various nanoparticulate delivery systems, polymeric NPs, solid lipid NPs (SLNs), metal NPs, liposomes, polymer-drug conjugates, and carbon-based nanomaterials are the most tested platforms in the veterinary area (Figure 1).

Overall, one of the main advantages of using nanocarriers for delivery of drugs in the livestock population is the reduced frequency of drug administration that results in reduced animal stress, lower treatment costs and lower number of visits by a veterinarian. For pets, the use of nanocarriers can contribute to improve the adherence to therapy, generally low as the animals are uncooperative [3]. In fact, of all veterinary applications, pets with cancer have benefited the most from nanocarriers, while farm animals have benefited much less because of the high cost of them. According to Chariou et al. [4], the Food and Drug Administration (FDA) has approved only a few microencapsulated products (including micro- and nanoscale carriers) for veterinary use, while about a dozen are under clinical trials, most of them for the treatment of canine diseases. This is because dogs are one of the most common pets in the world, and researchers generally agree that by studying diseases in dogs, it is possible not only to cure them but also to learn new opportunities for treating human diseases, performing comparative pathology or comparative oncology investigations. For these reasons, in this review, first a brief overview of the main nanocarriers used in veterinary medicine will be presented followed by an explanation of canine osteosarcoma (OSA) physiopathology considering its similarity to human OSA. A discussion of the different types of canine OSA treatment options will follow, including the benefits that nanocarrier-based drug delivery systems can provide to this important pathology.

## 2. Nanocarriers Used in Veterinary Medicine

Due to their peculiar physico-chemical properties, polymeric NPs have an undoubtedly potential for animals offering the possibility of more specific treatments. Feldhaeusser et al. assessed the in vitro effects of PEGylated PLGA-NPs loaded with a modified cisplatin for the treatment of canine brain tumors and found these NPs to be more effective and less toxic than the chemotherapeutic agent used as a reference [5]. Polymeric NPs were also used as a strategy to favor the healing process: in a recent study, Viswanathan et al. demonstrated the efficacy of a formulation containing NPs of calcium phosphate incorporated with chlorhexidine in the treatment of open wounds in animals [6]. The role of polymeric NPs is also crucial for vaccines. The PLGA-NPs encapsulated with the synthetic peptide BPI3V of bovine parainfluenza virus type 3 have been studied via intranasal route against the main respiratory diseases affecting calves and oxen. In comparison to empty NPs and BPI3V alone, they showed positive effects in the groups of mice tested [7].

Besides polymeric NPs, SLNs have been proposed for delivery of antibiotics with poor solubility to treat a variety of animal infections. For example, tilmicosin (a semi-synthetic antimicrobial agent) was entrapped in three different types of lipid NPs, which were then administered orally to broilers. Among the three tested systems, SLN-loaded tilmicosin showed the best results in terms of drug bioavailability and pharmacokinetic parameters [8].

Metal NPs (such as silver, gold, titanium, zinc, and copper NPs) have lately received increasing attention due to their potential in veterinary applications as well. Recently, silver NPs (AgNPs) have been studied by Yuan et al. who demonstrated their antimicrobial efficacy against multiple drug-resistant pathogens (*Staphylococcus aureus* and *Pseudomonas aeruginosa*) isolated from mastitis-infected goats [9]. Similarly, Fondevila et al. studied the effect of AgNPs (20 and 40 ppm) administered to weaned piglets. They found a substantial decrease in the number of ileal coliform bacterial colonies in treated pigs [10]. AgNPs were also found to be effective in treating experimental aflatoxicosis in broiler chickens [11], furthermore they were studied as adjuvants in rabies veterinary vaccines resulting free of side effects [12]. Interesting results have also been achieved with gold NPs (AuNPs), particularly when combined with glutathione-stabilized doxorubicin (DOXO) to treat feline fibrosarcomas. According to the findings, this innovative conjugate may be a powerful therapeutic agent capable of overcoming the resistance to DOXO by exhibiting high P-glycoprotein (P-gp) activity [13].

In recent decades, liposomes have also been studied to control the release of therapeutic compounds in animals [14]. Some studies reported the potential application of both non-PEGylated and PEGylated liposomes in pet animal cancer treatments [15]. Khanna et al. carried out an interesting in vivo study in dogs affected by spontaneous lung metastases demonstrating the safety and efficacy of an inhalant therapy based on interleukin-2 entrapped in liposome [16]. Some years later, M. L. Hauck and co-workers described a phase I trial in which a low temperature sensitive liposome formulation containing DOXO was used in combination with hyperthermia to improve drug delivery to solid tumors. They found that the treatment was well tolerated, and a favorable response profile was observed [17]. More recently, Withers et al. evaluated the effect of Lipocurc^®^, a liposome-encapsulated curcumin formulation, on the viability of canine OSA, mammary carcinoma, and melanoma cell lines. Their results indicate that Lipocurc^®^, compared to free curcumin (CURC), has certain inhibitory effects on cell viability. They also demonstrated the administration of Lipocurc^®^ infusion was feasible and well tolerated in cancer-bearing dogs [18].

Polymer-drug conjugates are another appealing drug delivery platform recently reported in veterinary literature. More specifically, chemical modification of sodium alginate with antibiotic gentamicin sulfate by carbodiimide chemistry has been proposed to create a water-insoluble antimicrobial material that is capable of killing microbes on contact, without the release of biocides. This type of antimicrobial material has proven to be very important in protecting surfaces from bacteria for long periods of time and could be employed for wound dressings and scaffolds for tissue engineering [19]. Another study relates to a combination of polymethacrylic acid and PEG evaluated for its effects on the intranasal administration of budesonide in rabbits. The such obtained pH-sensitive mucoadhesive copolymers were loaded with budesonide and then examined using various ethanol solutions. The results of administration of budesonide solution and budesonide-polymer in rabbits revealed the copolymer-drug conjugate adhered strongly to the nasal mucosa allowing for the high bioavailability of the drug [20]. Remaining in the field of polymers, dendrimers have also been explored as an approach for delivering drugs and active molecules in animals. They are monodispersed, and usually highly symmetric, spherical supramolecular structures with several advantages in veterinary medicine for the transport of various agents (e.g., genes and anticancer drugs) but also as tools for MRI imaging [21]. Moreover, due to their characteristics, they are particularly promising for the development of vaccines to prevent endemic infections [22]. For instance, Asgary et al. developed a nonlinear globular G2 dendrimer comprising citric acid and PEG 600 as an adjuvant in veterinary rabies vaccine. The results confirmed that this nanocarrier can enhance immune response since the in vivo assay showed a greater survival rate in the treated mice [23].

Carbon-based nanomaterials such as nanotubes, fullerenes and graphene have recently gained increasing interest as promising materials not only for drug delivery but also for bioimaging, biosensing, and tissue engineering applications. In particular, carbon nanotubes (CNTs), that are long and thin cylindrical carbon structure consisting of rolled-up sheets of single-layer carbon atoms (graphene), have been proposed in the veterinary area as an interesting tool for reproductive management, particularly for detection of the reproductive and fertility status of animals [24]. Indeed, it has been demonstrated that it is possible to detect estrus by implantation of nanotubes under the skin in animals to measure what level of estrogen hormones they have in their blood. This is due to the nanotubes binding with estradiol antibodies by fluorescence-producing signals that aid the reproduction monitoring system [25]. Moreover, functionalized CNTs have been demonstrated to be able to act as a carrier for a variety of therapeutic agents. As an example, the in vivo administration of CNTs-paclitaxel conjugate in a murine breast cancer model has been observed with higher efficacy in suppressing tumor growth and less toxic effects to normal organs [26]. However, despite the several obtained promising results of carbon-based nanomaterials, there are still tremendous opportunities to be explored and significant challenges and risks to be solved before their clinical applications.

A summary of the main nanocarriers used to prepare nanomedicines for veterinary application and discussed in this section is reported in Table 1.

## 3. Comparison of Canine and Human OSA

Canine OSA is a naturally occurring neoplasia that mimics, to a degree, the human counterpart: both demonstrate similar clinical characteristics, gross morphology, and histopathologic features, including the presence of microscopic tumors at the time of diagnosis, similar responses to conventional treatment schemes such as surgery and chemotherapy, and dysregulation of several key molecular pathways [27,28]. OSA is frequently diagnosed in the canine species, considering that 10,000 cases per year in the United States have been calculated [29]. The majority of canine OSA occur in the distal radius and proximal humerus: both sites are correlated to the maximum skeletal load of large or giant breed dogs, where strain, microtrauma and remodeling can occur [30,31].

Several authors have concluded that comparing the human and the canine species, valuable information on OSA etiopathology mechanisms can be obtained, with the ultimate aim of understanding whether a treatment proven to be effective in one species can be applied to the other [29,32,33].

It has been reported that OSA occurs in long bones of the appendicular skeleton near the metaphyseal growth plates, and the distal portion of the femur and the proximal tibia are the most affected sites in humans [34]. In humans, OSA appears primarily in adolescence, with most cases between the ages of 10 and 14, suggesting a close relationship between early pubertal rapid bone growth and tumor development [35] with a higher risk in taller children [36].

Several factors contribute to the development of OSA in dogs and humans, including environmental exposures, gene mutations, and specific predispositions. As an example, individuals with Li-Fraumeni Syndrome, who carry germline mutations in the tumor suppressor gene p53 or those harboring the oncogenic variant RB1, may develop secondary OSA after radiation or chemotherapy [30]. The importance of tumor suppressor genes has been well characterized in humans while in veterinary counterpart is under investigation: a genomic analysis of Greyhounds, Rottweilers, and Irish Wolfhounds has revealed 33 inherited risk loci, including one upstream of CDKN2A/B that can disrupt the critical balance between cellular proliferation and senescence promoting tumorigenesis [37]. Accordingly, CDKN2A deletion and subsequent loss of p16 expression has been observed in humans with OSA [38]. Other breeds including the Scottish Deerhound, Great Dane, and St. Bernard, have been shown to be hereditary predisposed, but involved genes have not yet been identified [39].

Both in human and canine species, the primary tumor’s biological behavior demonstrates a local aggression, visible in bone lysis alone or associated with new bone proliferation: the pure lytic OSA is macroscopically soft with colored areas of hemorrhage and necrosis, while productive tumors are gray and firm due to the presence of osteoid or cartilage derived from OSA [40]. Metastasis can be identified in lungs, other bones, soft tissues, lymph-nodes, and visceral organs [34,41]. The traditional treatment approach is a limb sparing procedure followed by chemotherapy which can consist in a single or combined administration of DOXO, cisplatin or carboplatin, and methotrexate [42,43].

Based on the microscopic morphology, several histological patterns can be described. According to the World Health Organization’s histologic classification of tumors of domestic animals, several similarities can be drawn between six types of canine OSA, and the eight types described for humans [44]. Furthermore, the comparison of the different histological types of OSA between canines and humans has permitted to understand that although six different subtypes of OSA have been identified in dogs (i.e., osteoblastic, chondroblastic, fibroblastic, telangiectatic, giant cell-rich, and poorly differentiated types), they are similarly expressed in humans [45].

Nevertheless, considering only the clinical aspects related to the veterinary side, histopathological heterogeneity is a complicating factor both in diagnosis and prognosis, thus leading to variable therapeutic success [46]. To improve the accuracy of the diagnosis, other supporting factors can be considered: elevated serum alkaline phosphatase, staining of cytologic specimens and immunohistochemistry can be helpful in distinguishing OSA [47] from other sarcomas [40].

After diagnosis, standard treatment procedures mainly involve surgical removal of the tumor followed by chemotherapy. In canine patients, in contrast to humans, limb amputation and limb-sparing are commonly performed, leading to a median survival time of one year. Dogs can receive simple or associated chemotherapy, although a multiagent approach has not been shown to be significantly superior to carboplatin [48].

To collect a huge quantity of information about this type of tumor, different animal models have been considered. The mouse model has been widely used and has the advantage of offering a homogeneous population that is easy to be manipulated to obtain specific genetic and molecular patterns, rapidly developing primary and metastatic tumors [34]. Nevertheless, it is not a spontaneous condition, and this could be a bias in comparison with natural occurring neoplasia. Canine OSA is considered the best model of pediatric and adolescent OSA [28]. On the other hand, companion dogs can demonstrate a heterogeneous presentation of OSA that may at first be difficult to understand, but that can truly represent the varied situation of the human population [34]. Some experimental settings have been designed using dogs as models to investigate the implications of different accidental human exposure. It was noted that several types of sarcomas, including OSA, may develop in humans exposed to radiation or radiation therapy just as in animals [49]. It was also observed that OSA caused by plutonium in beagle dogs mimics OSA caused by plutonium in humans, with most lesions located in the axial skeleton [50].

## 4. Potential Utility of Canine OSA as a Human OSA Model

In vitro/in vivo model-based comparative oncology studies are necessary to supplement the scientific knowledge acquired from clinical cases. In recent decades, mouse and canine models have been widely used to evaluate new diagnostic tools and therapeutic strategies that can be beneficial for humans. Canine immortalized cell lines are currently commercialized by international companies or shared among researchers. As explained in the previous section, canine OSA can be classified in six different types according to the histological pattern and can demonstrate different biological features: related to this, several cell lines have been developed from primary or metastatic sites [51]. Moreover, it is possible to begin a primary cell line starting from biopsies or surgical specimens that can offer new information derived from clinical site, permitting testing of new drugs, and understanding biological behavior of non-immortalized OSA cells [34].

Clinical trials on companion dogs with OSA allow to collect practical information and are generally classified into four different types: cancer biology, drug development, diagnostics, and imaging. Trials should not be intended only as experimental models because dogs are increasingly considered family-members, thus trials can be structured to mirror their pediatric counterparts or specifically designed to answer a veterinary-specific question or goal. The demand for improved diagnostic tools and therapeutic approaches in veterinary medicine has been significantly increased in recent years, leading to valuable advances in treatment, prognosis, and improvement of quality of life of veterinary patients. Even though clinical trials are designed to collect information from human counterparts, ethical considerations must always be considered: serial bone tumor biopsies, for example, should be avoided, as they may induce additional pain and may predispose to pathological fracture [34].

In recent years, companion dogs affected by cancer have become increasingly important for testing the tolerability, pharmacology, and pharmacodynamic effects of novel anticancer treatments [52]. Metastatic OSA is a serious issue that continues to be unmet in human research and could be addressed by studying dogs. The localization and the spread of metastasis is similar between the two species and can be placed in lung (50−85%), distant bone, regional lymph nodes, liver, and skin layers [41,53]. Considering only pulmonary metastasis, they are commonly removed in human medicine, while lung metastasectomy is not often performed in veterinary medicine due to the rapid metastatic progression. Turner and colleagues reported that metastasectomy was associated with an increase in survival time in dogs affected by stage III OSA, and those presenting less than three lung metastases at the thoracic x-ray underwent to surgical removal and demonstrated a significantly longer disease-free interval (more than 275 days) compared to untreated ones (49 days) [54].

Certainly, dogs are more reliable models for assessing the toxicity of novel therapies than rodents are. Like humans, canine patients can be monitored as required for hematological and biochemical toxicities through routine clinical pathology and sophisticated monitoring (e.g., 24 h continuous electrocardiographic telemetry, continuous blood pressure measurement, ophthalmologic monitoring, echocardiography, gait analysis, advanced imaging). Companion animals also receive supportive care (e.g., antiemetics, antidiarrheals, antibiotics, etc.) as it is for human patients [52]. Moreover, to facilitate the sharing of information about genome sequencing of human and canine cancers in the scientific community, the Integrated Canine Data Commons has been created to improve comparative cancer research. However, note that although the histological and biological features of human and canine cancer cells are quite similar, their response to treatments may be totally different [55,56].

## 5. Canine OSA Treatments

### 5.1. Conventional Therapies

A primary treatment strategy for OSA includes local control (surgery) and systemic control (chemotherapy and radiotherapy). The surgical approach, mainly limb amputation, was established to be the care for local management of canine OSA since the 1970s and it is still considered the standard treatment to definitively treat the neoplasia, in association with chemotherapy to delay metastasis [57,58]. The main advantages induced by this technique are the elimination of pain and the avoidance of pathological fracture. Even if the aesthetic result might be shocking at first, the functional result is good even in large breed dogs in which this neoplasia is frequently diagnosed [59,60]. Each case should be carefully and globally evaluated to understand if a dog might be a good candidate for surgery: amputations can be contra-indicated by severe obesity, concurrent orthopedic or neurological conditions, metastatic disease, and non-compliance of owners [61]. In these cases (or when owners refuse amputation), a limb sparing surgery can be performed or radiation could be applied, alone or in combination with analgesics administration, with the final aim to promote a good quality of life and reducing tumor-related clinical signs [57,61,62].

The surgical techniques to preserve the limb encoded the resection of the part where OSA is identified and clean margins: the gap can be filled with a frozen cortical allograft, an endoprosthesis or with the resected neoplastic bone after its pasteurization, autoclave sterilization or irradiation [63,64,65]. However, surgery alone is considered to be palliative: no statistical difference in survival time has been demonstrated between amputation and limb-sparing surgery if adequate systemic chemotherapy is given and similar findings have also been reported in humans with limb-sparing surgery. Surgery and/or radiotherapy can help to improve survival in dogs with OSA when chemotherapy is administered as an adjuvant. Chemotherapy regimens include DOXO, cisplatin, carboplatin and lobaplatin administered alone or in combination. In this condition, the median survival time increases from 103–175 days to 262–450 days [57]. Bisphosphonates have also been tested alone or in association with palliative- intent radiotherapy: bisphosphonates are synthetic analogs of pyrophosphate with a high affinity for bone material and are able to inhibit osteoclast activity, limiting osteolysis and primary bone lesions [66]. Data reported in a retrospective study suggested that no difference could be found in dogs treated with radiotherapy, with or without the concomitant administration of bisphosphonate [61].

It has been demonstrated that early postoperative chemotherapy has no substantial benefits; therefore, it is preferable to wait for the patient to recover from surgery and heal the surgical wound. Furthermore, chemotherapy is usually less effective in the presence of macroscopic metastatic disease [57].

Radiotherapy has been proposed and evaluated with several aims: on one hand, curative-intent local treatment of canine appendicular OSA while on the other, the palliative intent. Several studies have evaluated the concurrent administration of chemotherapy with palliative-intent radiotherapy showing conflicting results about improvement of survival times [67,68]. Moreover, some pathological fractures after irradiation have been recorded [57]. An intra-operatively single fraction of 70 Gy irradiation given after exteriorization of the tumor bone segment has been used in combination with chemotherapy highlighting the possibility to cause complications in 69% of cases, thus reducing the possibility to apply this technique to a limited number of dogs [64,69].

### 5.2. Innovative Therapies

In addition to conventional therapies, some innovative strategies have been investigated in canine OSA over decades to increase efficacy of OSA treatment and reduce metastasis. Several authors have proposed the association of a drug with potential cytotoxic and/or anti-metastatic activity to standard chemotherapy treatment. As an example, Kozicki et al. investigated the combination of carboplatin with pamidronate, a nitrogen containing bisphosphonate antiosteoporosis agent, which was found to show high cytotoxicity against OSA cell lines. Unfortunately, the results of the study revealed that adding pamidronate to carboplatin chemotherapy for treatment of canine OSA, although safe, did not affect the efficacy of the treatment [70]. Similarly, the results achieved by enriching carboplatin with gemcitabine, a nucleoside analogue, used as chemotherapeutic agent were comparable to those obtained with carboplatin monotherapy, without significant improvements [71]. Another study was performed on 303 dogs to evaluate whether adding BAY 12–9566, a matrix metalloproteinase inhibitor able to suppress metastasis ability of tumor cells, can improve OSA treatment based on DOXO followed by limb amputation. Results showed that this combined therapy did not positively influence the survival time as the median survival time in both groups was less than 8 months [72].

In another study, the combination of DOXO with suramin (polysulfonated naphtylurea), a non-cytotoxic substance able to increase the chemosensitivity of tumors in vitro, was investigated. Results obtained with this combined therapy administered after amputation in dogs with OSA were similar to monotherapy [73]. Sometime before, Kurzman and associates showed that the advantage of adding liposome-encapsulated muramyl tripeptide phosphatidylethanolamine (L-MTP-PE) to standard therapy (with cisplatin and surgery) was the survival time. In particular, they noted that the concomitant administration of L-MTP-PE with cisplatin chemotherapy and surgery did not yield any survival advantages. However, its addition after cisplatin significantly increased survival time (14.4 months) when compared to other groups treated with surgery, cisplatin, and liposomes alone (9.8 months). In addition, these dogs also had a significantly longer metastasis-free interval compared with dogs treated with placebo liposomes [74].

Immunotherapy is another alternative option to conventional therapies for canine OSA. Tumor growth in dogs with OSA has been hypothesized to be influenced by humoral factors found in their serum (such as blocking antibodies or antigen-antibody complexes) since the 1970s. Over time, preclinical and clinical evidence supported the concept that OSA is an immunogenic tumor and that it can potentially be controlled using therapy able to modulate the response of the immune system. For these reasons, novel immunotherapeutic strategies have been widely explored ranging from monoclonal antibodies to therapeutic vaccination and from cytokine therapy to activation of innate immune cells [75].

As an example, D.M. Haines and co-workers demonstrated by immunohistochemical staining the ability of monoclonal antibodies TP-1 and TP-3 to bind to neoplastic OSA canine tissue, meanwhile limited staining was found in a wide range of normal canine tissues [76]. Biller et al. demonstrated the prognostic role of the regulatory T-cells in dogs with OSA. Namely, they found that dysregulation of regulatory T-cells was associated with decreased survival in dogs with osteosarcoma [77]. Other authors found a significant increment of circulating myeloid-derived suppressor cells in dogs with OSA compared to normal dogs [78]. More recently, a dermal peptide-based anticancer vaccine was developed, and its efficacy was evaluated in a clinical trial in dogs with non-metastatic appendicular osteosarcoma. Two groups of dogs were enrolled in this study: one group received standard treatment, consisting in limb amputation and chemotherapy, and the other group received additional vaccination. The second group demonstrated a significant increase of the period prior to developing metastasis and of survival [79].

Unfortunately, many of the immunotherapies currently being studied have shown limited ability to significantly prolong survival time over standard therapies or are still in the preliminary stages of testing. Nonetheless, continued research in how to best manage OSA disease remains a highly desirable strategy that holds promise both in dogs and human beings. Recently, the involvement of kinases in canine OSA, often overexpressed or dysregulated, suggested a consequent potential of kinase inhibitors to treat this disease. Mauchle et al., for example, investigated in vitro the ability of 80 kinase inhibitor compounds to inhibit the proliferation of four canine OSA cell lines. Four protein kinase inhibitors were identified with broad antiproliferative activity, and these compounds also enhanced DOXO activity [80]. Similarly, it has been discovered that the overexpression of platelet derived growth factor receptors (PDGFRs), that are tyrosine kinase receptors, and their specific ligands, play a crucial role in the growth and progression of canine OSA. As a result, they may represent suitable targets for specific (targeted) OSA therapy [81].

The emerging knowledge of the pathogenesis and genetic abnormalities associated with OSA have paved the way for a third innovative approach in the treatment of OSA that is gene therapy. Several gene therapy strategies using both viral and non-viral vectors have been developed over the last twenty years.

The most important gene therapy approach for OSA involved the mechanism of mutation compensation based on the idea that the disease is caused by a single genetic alteration such as loss of the retinoblastoma (Rb) gene or a mutation in p53 gene. Consequently, Rb and p53 have been examined as possible targets for gene therapy in OSA. Accordingly, a series of OSA cell lines (MG-63, K-HOS, SJSA-1, and SaOS-2) were transfected with wild-type Rb via adenoviral vector, resulting in a reduction of their proliferation [82]. Similarly, Oshima et al. tested adenoviral vectors containing p53 analogues (p73 and p63) in OSA cell lines and found they improved apoptosis both in vitro and in vivo [83].

However, cancer, unlike other diseases, is caused by a cascade of genetic abnormalities (rearrangements, deletions, frame shift mutations, etc.), thus alternative gene therapy approaches that are less dependent on the genetic background of the target cell populations have been developed, such as suicide gene therapy and immune-potentiation gene therapy.

Gene therapy based on suicide genes converts non-toxic prodrugs into toxic compounds through a transduced cell’s gene product whereas immunopotentiation gene therapy aims to achieve anti-tumor immunity through two basic approaches: (i) increasing the ability of the immune system to recognize tumor cells and (ii) enhancing innate immune system efficiency [84]. A general boost of the immune system can be achieved through the introduction of genes coding for cytokines and other co-stimulatory molecules, such as genetically modified T-lymphocytes that recognize tumor cells more effectively [85]. Indeed, some studies have shown that the transduction of OSA cells with the cytokine IL-12 gene, intranasal administered, reduced the ability of these cells to form lung metastases in nude mice [86]. Although the mechanisms of IL-12 anti-tumor activity are not fully understood, they may be linked to both its ability to inhibit angiogenesis and stimulate T-cells and NK cells. Adenovirus-mediated B7-1/Fas chimeric gene transfer has also been reported to activate T-cell activation and induce apoptosis of osteosarcoma cells [87].

**Figure 2:**
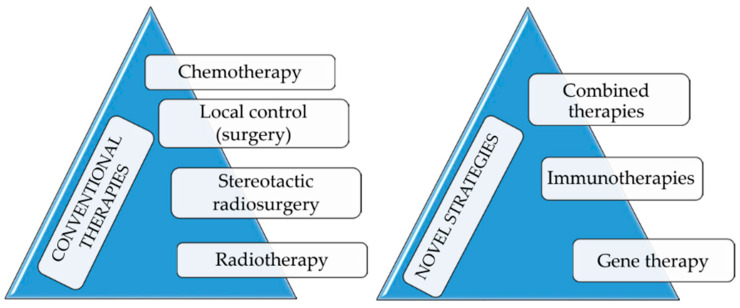
Conventional and innovative approaches to canine OSA treatment.

It is noteworthy that gene therapeutic approaches for treating OSA have demonstrated promising outcomes even though additional evaluations are warranted. At present, what we can say is that it is possible to achieve more complete disease control by combining gene therapy with conventional treatments. Anyway, and notwithstanding the above, this review is not intended to provide a comprehensive discussion of all these innovative strategies which are summarized in Figure 2.

## 6. Nanocarriers for Drug Delivery in Canine OSA Treatments

Despite many efforts to develop new strategies, nowadays the primary OSA therapy continues to be based on chemotherapy. As mentioned earlier, as for other tumors, chemotherapy is employed for OSA as a palliative care, as a pre-surgery option to reduce tumor size, or as a post-surgery treatment to prevent disease recurrence. However, chemotherapeutic agents can have many side effects, and in many cases, their effectiveness is hampered by the phenomenon of multi-drug resistance (MDR). On the other hand, evidence has been obtained that by incorporating chemotherapeutic agents into nanocarriers, these issues can be overcome. In fact, nanoscale drug delivery systems can target specific tissues or malignant cells reducing the occurrence of toxic effects. Moreover, they serve as multifunctional platforms to overcome cancer MDR for example by helping drugs to escape from the recognition of pump transporters in MDR cancer cells resulting in increased therapeutic efficacy.

### 6.1. Nanoparticles

Nanoparticles have been investigated as delivery systems of important anticancer agents such as paclitaxel (PTX) characterized by a broad spectrum of activity. This is because, although PTX is considered highly effective in the treatment of many cancers, it is poorly used in dogs because co-solvents required for its solubility can induce an acute hypersensitivity. Consequently, some authors have developed a formulation of nanoparticulate PTX (CTI 52010) consisting of the drug and normal saline. In this study, 120 mg/m^2^ was the maximum tolerated dose, and neutropenia of grade 4 was the dose-limiting toxicity. Higher doses resulted in gastrointestinal toxicity grades 1–2. No signs of organ toxicity (liver, kidney, spleen) were observed at post-mortem examination. In summary, CTI 52010 was well tolerated by normal dogs when administered intravenously [88].

Other authors have reported the use of Pam-DOXO-NPs in dogs with OSA, which are DOXO-loaded polylactide NPs coated with bone-seeking pamidronate (Pam) for the targeted treatment of malignant skeletal tumors. They reported that the repeated administration of Pam-DOXO-NPs in dogs with natural OSA was not associated with any hematologic, non-hematologic, or cardiac toxicities. A nuclear scintigraphy study showed that Pam-DOXO-NPs could target malignant bone and exert measurable anticancer functions, as assessed by histopathological analysis of percent tumor necrosis [89].

Recently, Ulutas et al. developed chitosan NPs loaded with clinoptilolite a natural, non-toxic zeolite that exhibits ion exchange and adsorbent properties and has biological functions such as antiviral, antibacterial, anti-inflammatory, antidiabetic, and anticancer activities. Briefly, clinoptilolite was converted into a NP by encapsulation with chitosan by spray-drying. Different doses of NPs were administered to canine OSA cells, and it was found that they effectively and rapidly decreased cell viability by exerting a pro-apoptotic effect. Moreover, clinoptilolite NPs showed this effect at much lower doses than in previous studies in which it was used in its pure form [90].

An in-depth study by Malek et al. examined the interactions between DOXO conjugated with glutathione-stabilized gold nanoparticles (Au-GSH-DOXO) and P-gp activity in the D17 canine OSA cell line. The human OSA cell line U2OS is sensitive to DOXO and was used as a negative control. As compared with free DOXO, Au-GSH-DOXO displayed a greater cytotoxic effect on D17 but not on U2OS (IC50 values for Au-GSH-DOXO versus DOXO were 7.9 g/mL versus 15.2 g/mL respectively). No toxicity was observed for either cell line at any concentration of Au-GSH (10–1000 g/mL). According to this study Au-GSH-DOXO may be considered a valuable treatment for canine OSA being able to bypass P-gp pathways [91].

Continuing discussion on the delivery of DOXO, Chirio et al. developed calcium phosphate coated lipid NPs (CaP-NPs) loaded with a lipophilic ester of DOXO and tested them for their impacts on drug uptake and cytotoxicity into human and canine OSA cells. Results were very similar on both the cell lines showing an increase in drug uptake and cytotoxicity for CaP-NPs, especially when calcium ions were externally exposed, which paves the way for future applications in both human and veterinary medicine [92].

### 6.2. Liposomes

In addition to NPs, liposomes have been extensively studied as drug delivery systems for anticancer agents due to their ability to target cancer cells and reduce the negative side effects of free cytostatic drugs.

One of the first literature reports on the administration of liposomes in dogs for the treatment of OSA was that of Shi et al., who showed that the previously mentioned L-MTP-PE, muramyl tripeptide phosphatidylethanolamine encapsulated in liposomes, effectively delayed, or prevented, metastasis in dogs with spontaneous osteosarcoma in a randomized clinical trial. They investigated the in vivo effect of DOXO administered alone and in combination with L-MTP-PE on monocyte activation and tumor necrosis factor activity in serum. They found that DOXO in combination with L-MTP-PE enhanced the activation of monocytes triggered by DOXO or L-MTP-PE alone in dogs and suggested that a combination of DOXO and L-MTP-PE could be successful in the early treatment of cancer patients [93]. The same authors treated dogs suffering from spontaneous appendicular osteosarcoma with cisplatin chemotherapy and studied the efficacy of L-MTP-PE in preventing or delaying the appearance of metastases. L-MTP-PE, when administered after amputation, exerted an antimetastatic effect, while no survival benefit was found when L-MTP-PE was administered concurrently with cisplatin chemotherapy [74].

A few years later, Vail and coworkers described the intravenous administration of Doxil^®^, PEGylated liposomes containing DOXO, to a group of 51 dogs with measurable tumors of various histological types and locations, which received a total of 103 Doxil^®^ treatments every 3 weeks at a dosage of 0.75–1.1 mg/kg. Doxil^®^ was well tolerated at doses comparable to those of free DOXO in tumor-bearing dogs. In both human and canine OSA cells, CaP-NPs increased drug uptake and cytotoxicity, particularly when exposed to calcium ions externally, which could lead to future veterinary and human applications. An overall response rate of 25.5% was observed, with 5 dogs responding completely and eight responding partially to treatment. Only 4 dogs were affected by OSA and unfortunately none of them experienced significant tumor reduction. Nevertheless, the results of this study need to be carefully evaluated due to the small number of animals in each tumor group [94].

The same authors tested a PEGylated liposomal formulation of cisplatin (SPI-77) and obtained liposomes with a longer circulation time in blood, a higher area under the time-concentration curve (AUC) and a delayed plasma clearance. Forty domestic dogs with spontaneously occurring OSA were randomized to receive SPI-77 (350 mg/m^2^ intravenously every 3 weeks for four treatments) or carboplatin (300 mg/m^2^ intravenously every 3 weeks for four treatments) along with amputation of the affected limbs. Loading SPI-77 with cisplatin allowed safe administration of a dose five times higher than the maximum tolerated dose of free cisplatin in dogs. However, median disease-free survival and overall survival were not significantly prolonged, although there was a long-term increase in disease-free survival compared to dogs receiving free carboplatin [95].

Some years later, Ichihara et al. studied the dose dependence and effects of repeated administration of PEGylated liposome on the induction of accelerated blood clearance (ABC). The ABC is known to be related to the abundant production of anti-PEG IgM in response to the first dose of PEGylated liposomes. As a result of this study, it was noted that PEGylated liposomes lose their long-circulating properties when they are administered repeatedly at certain intervals to the same animal. Moreover, they noted that in mice and rats, the spleen is mainly involved in the secretion of anti-PEG IgM [96]. The same group of researchers observed that in Beagle dogs the ABC phenomenon was affected by the addition of empty PEGylated liposomes during sequential administration of Doxil^®^ suggesting the importance of pre-clinical studies in developing this type of systems [97].

More recently, Withers et al. developed Lipocurc^®^, a particular formulation of CURC encapsulated in liposomes. The use of CURC has been shown to inhibit cancer in vitro, but its poor bioavailability due to low water solubility, suboptimal absorption by tissues and rapid metabolism and excretion compromises its therapeutic potential. The effect of Lipocurc^®^ compared to free CURC on the viability of canine OSA, melanoma and mammary carcinoma cell lines was investigated. The ability of Lipocurc^®^ to inhibit endothelial cell viability, migration and formation was also evaluated. In addition, a pilot clinical trial consisting of four weekly 8-h Lipocurc^®^ infusions in 10 cancer-bearing dogs was performed. The results of in vitro experiments indicate that Lipocurc^®^ inhibits the viability of canine cancer cell lines and the in vivo trial shows that Lipocurc^®^ at high concentrations can stabilize the disease in OSA-bearing dogs [18].

Liposomal formulations have also been proposed for diagnostic purposes: a long-circulating liposomal iodine contrast agent (Liposomal-I) was developed for computed tomography imaging of solid tumors in domestic dogs with naturally occurring cancer. It was found that Liposomal-I significantly enhanced the visualization of the vascular compartments facilitating the identification of primary and metastatic liver tumors. In particular, after 24 h they found an improved pattern scan allowing identification of extra-hepatic, extra-splenic tumors, including histiocytic sarcomas, anaplastic sarcomas, breast carcinomas and lung tumors. In addition, this study showed that the contrast agent is subjected to non-renal, reticulo-endothelial systemic clearance [98].

### 6.3. Miscellaneous

Besides liposomes and NPs, other types of drug delivery systems have been proposed to improve canine OSA treatment. As an example, recently, some authors developed a novel peptide-based nanofiber precursor (NFP) capable of exploiting the leaky tumor neovasculature for promoting drug delivery after parenteral administration. They loaded NFP with aldoxorubicin, which is an albumin-bound prodrug of DOXO and tested it on canine osteosarcoma (HMPOS, D-17, Abrams) cell lines. They found that inhibitory concentration (IC50) was lower than both free aldoxorubicin or DOXO, indicating that drug-loaded NFPs are cytotoxic for various canine OSA cell lines in vitro [99].

Horise et al. carried out a clinical trial using an anticancer micelle of 60–70 nm called NC-6300, employed as a sonosensitizer in sonodynamic therapy, a minimally invasive cancer therapy that uses a chemical sonosensitizer and high-intensity focused ultrasounds (HIFU). In this study, NC-6300 was loaded with the drug epirubicin. They noted that due to the enhanced permeability and retention effect, NC-6300 preferentially accumulated in tumor cells and efficiently induced ROS generation further increasing the effectiveness of the sonodynamic therapy. Furthermore, no adverse events were observed in the four treated dogs affected by spontaneous tumors, including OSA, hepatocellular carcinoma, and prostate cancer. Overall, this trial supported the usefulness of a such approach combining a low dose of NC-6300 and low-energy HIFU which allows a reduction in drug dose and ultrasound irradiance compared to conventional monotherapies [100].

In a case report, some researchers subjected an 8-year-old male castrated hound with left distal ulnar osteosarcoma to limb-sparing ulnectomy with local adjunctive carboplatin in a poloxamer copolymer gel (poloxamer 407). Poloxamer 407 can thicken to a gel when warmed to temperatures higher than 25 °C. No local or remote adverse effects were noted from the local delivery system of carboplatin in poloxamer 407. The case in the report developed local recurrence at 296 days after ulnectomy and distant metastases. These preliminary results, even if further efficacy studies are needed, revealed that poloxamer 407 might represent an easy and safe method for local adjunctive therapy after tumor resection [101].

Previously, Withrow et al. investigated the effect of a biodegradable cisplatin containing implant (OPLA-Pt) into the wound adjacent to the allograft used to reconstruct the osseous defect after limb-sparing radius and/or ulna surgery in OSA-bearing dogs. The results of this trial evidenced reduced rate and increased time of local recurrence. The reduced rate of local recurrence obtained upon OPLA-Pt application was not statistically significant, however a trend was observed. Furthermore, dogs in the treated group were nearly half as susceptible to development of local recurrence than untreated dogs employed as reference [102].

A detailed summary of the main nanocarriers employed in the treatment of canine OSA is reported in the following Table 2.

## 7. Conclusions

This review provides an overview of the role of nanocarriers as innovative tools in the treatment of veterinary tumors, with specific reference to canine OSA.

Improving the quality of life and survival time of dogs suffering from OSA represents an important achievement, both for its scientific relevance in the field of innovative drug delivery, and for its social aspects due to the unique dog-human relationship that results in dogs being considered as an extension of the family.

In recent years, nanocarriers have been increasingly explored in the veterinary field to solve most limitations to conventional treatments of animal diseases. Although the demand is growing, currently, very few nanoscale delivery systems have been approved for clinical application, owing to high costs of development and experimental trials.

Regardless, platforms mediated by nanocarriers applied to overcome the widespread failures of conventional chemotherapies, are worthy of further studies. In addition, because human and canine OSA are similar, the outcomes in this field might open new opportunities in human medicine.

## Figures and Tables

**Table 1:** Studies describing the use of nanocarriers in veterinary medicine.

Nanocarrier	Drug	Disease/Utility	Results	Reference
PEGylated PLGA-NPs	Modified cisplatin	Canine brain tumors	More effective and less toxic than reference	[5]
Calcium phosphate NPs	Chlorhexidine	Wounds in animals	Enhanced wound healing compared to control samples	[6]
PLGA-NPs	Synthetic peptide BPI3V of bovine parainfluenza virus type 3	Respiratory diseases of calves and oxen	More positive effects compared to empty NPs and BPI3V alone	[7]
Lipid NPs	Tilmicosin	Animal infections	SLNs showed the best results in terms of drug bioavailability and pharmacokinetic parameters	[8]
Silver NPs		Drug-resistant pathogens in goats	Antimicrobial efficacy against multiple drug-resistant pathogens	[9]
		Ileal coliform bacterial in pigs	Decrease in the number of ileal coliform bacterial colonies	[10]
		Aflatoxicosis in broiler chickens	Effective in treating alflatoxicosis	[11]
		Adjuvants in rabies vaccines	Free of side effects	[12]
Gold NPs	Glutathione-stabilized DOXO	Feline fibrosarcomas	Capable of overcoming the resistance to DOXO by exhibiting high P-glycoprotein activity	[13]
Liposomes	Interleukin-2	Spontaneous lung metastases in dogs	Safety and efficacy	[16]
	DOXO in combination with hyperthermia	Solid tumors	High tolerability and favorable response profile	[17]
	CURC	Cytotoxicity on canine cancer cell lines	Inhibitory effect on cell viability. Feasible and well tolerated administration via infusion	[18]
Polymer-drug conjugates	Gentamicin-sodium alginate	Anti-microbial activity	Protection of surfaces for long periods; employable for wound dressings and scaffolds for tissue engineering	[19]
	Budesonide	Respiratory disorders in rabbits	Slow release and high bioavailability of drug	[20]
Dendrimers	Citric acid and PEG 600	Rabies vaccine	Enhanced immune response	[23]
Carbon nanotubes	Paclitaxel	Murine breast cancer	High treatment efficacy and minimum side effects	[26]

**Table 2:** Studies describing the use of nanocarriers in canine OSA treatments.

Substance	Nanocarrier	Study Results	Reference
DOXO	Polylactide NPs coated with pamidronate (Pam-DOXO-NPs)	Ability to target malignant bone and measurable anticancer activities	[89]
	Glutathione-stabilized gold NPs (Au-GSH-DOXO)	Au-GSH-DOXO, compared to free DOXO, presented a greater cytotoxic effect on D17 by bypassing P-gp	[91]
	PEGylated liposomes (Doxil^®^)	None of the four dogs affected by OSA showed significant tumor reduction.	[94]
DOXO ester	Calcium phosphate coated lipid NPs	Loading DOXO in CaP-NPs allowed increased cellular uptake and cytotoxicity both in human and in canine OSA cell lines	[92]
Clinoptilolite	Chitosan NPs	Nanoclinoptilolite decreased cell viability and induced caspase-3- and -7-mediated apoptosis in treated canine OSA cells.	[90]
Muramyl tripeptide phosphatidylethanolamine (MTP-PE)	Liposomes	L-MTP-PE, after amputation, exerted an antimetastatic effect. No survival benefit when administered concurrently with cisplatin	[74]
Muramyl tripeptide phosphatidylethanolamine (MTP-PE)	Liposomes	DOXO in combination with L-MTP-PE enhanced the activation of monocytes triggered by DOXO or L-MTP-PE alone in dogs	[93]
Cisplatin	PEGylated liposomes (SPI-77)	SPI-77 allows the safe and repeated delivery of doses up to five times the maximally tolerated dose of native cisplatin in OSA bearing dogs	[95]
CURC	Liposomes (Lipocurc^®^)	Lipocurc^®^ in vitro inhibited the viability of canine cancer cell lines and in vivo showed ability in stabilizing the disease in OSA-bearing dogs	[18]
Iodine	Liposomes (Liposomal-I)	The long circulating Liposomal-I contrast agent enabled prolonged visualization of small and large tumors in companion dogs with naturally occurring cancer.	[98]
Aldoxorubicin	Nanofiber peptide (Aldoxorubicin-NFP)	The IC50 for aldoxorubicin-loaded NFP was lower than free aldoxorubicin or doxorubicin in OSA cell lines	[99]
Epirubicin	Anticancer micelles (NC-6300)	Antitumor efficacy was achieved through the combination of the anticancer NC-6300 micelle and HIFU.	[100]
Carboplatin	Poloxamer 407 gel	No wound healing complications or adverse effects occurred after local use of carboplatin in poloxamer 407. The local recurrence-free interval was 296 days from surgery, and the survival time was 445 days from initial diagnosis.	[101]
Cisplatin	Polylactic acid implant (OPLA-Pt)	OPLA-Pt reduces the rate of local recurrence after limb-sparing surgery in dogs with OSA. Furthermore, systemic toxicity associated with cisplatin release from OPLA-Pt is minimal.	[102]

## Data Availability

Not applcable.

## Abbreviations

OSA	osteosarcoma
NPs	nanoparticles
SLNs FDA	solid lipid NPs Food and Drug Administration
AgNPs	silver NPs
AuNPs	gold NPs
DOXO	doxorubicin
CURC	curcumin
CNTs	carbon nanotubes
L-MTP-PE	liposome-encapsulated muramyl tripeptide phosphatidylethanolamine
PDGFRs	platelet derived growth factor receptors
MDR	multi-drug resistance
PTX	paclitaxel
CTI 52010	formulation of nanoparticulate paclitaxel and normal saline
Pam	pamidronate
Au-GSH-DOXO	DOXO conjugated with glutathione-stabilized gold nanoparticles
P-gp	P-glycoprotein
CaP-NPs	calcium phosphate coated lipid NPs
SPI-77	STEALH liposome-encapsulated cisplatin
ABC	accelerated blood clearance
NFP	peptide-based nanofiber precursor
NC-6300	anticancer micelle of 60–70 nm
HIFU	high-intensity focused ultrasounds
OPLA-Pt	biodegradable polylactic acid-cisplatin containing implant
